# Associations of the triglyceride-glucose index, triglyceride glucose-body mass index, waist-triglyceride index and modified triglyceride-glucose indices with mortality in cardiovascular-kidney-metabolic syndrome stages 0–4: Evidence from NHANES 1999–2020

**DOI:** 10.1515/jtim-2026-0014

**Published:** 2026-02-13

**Authors:** Jingya Zhao, Xinning Lu, Hui Wang, Qin Chen, Yigang Wan

**Affiliations:** Department of Traditional Chinese Medicine, Nanjing Drum Tower Hospital Clinical College of Nanjing University of Chinese Medicine, Nanjing, Jiangsu Province, China; Department of Nephrology, Tongde Hospital of Zhejiang Province, Hangzhou, Zhejiang Province, China; Department of Traditional Chinese Medicine, Nanjing Drum Tower Hospital, Affiliated Hospital of Medical School, Nanjing University, Nanjing, Jiangsu Province, China; Institute of Chinese Medicine, Nanjing University, Nanjing, Jiangsu Province, China

## To the editor

Cardiovascular-Kidney-Metabolic (CKM) syndrome, introduced by the American Heart Association (AHA) in October 2023, reflects the interconnected progression of cardiovascular disease (CVD), chronic kidney disease (CKD), and metabolic abnormalities.^[1]^ Affecting more than 90% of US adults, CKM syndrome is associated with increased risks of cardiovascular events, CKD and mortality, particularly in stages 3–4 where mortality exceeds 188.8 deaths per 1,000 person-years.^[2,3]^ Given its high prevalence and poor prognosis, effective strategies are urgently needed to prevent CKM-related adverse outcomes. Insulin resistance (IR), characterized by impaired tissue responsiveness to insulin, plays a critical role in CKM development and progression. Several studies suggest that the triglyceride-glucose index (TyG), TyG-body mass index (TyG–BMI), waist-triglyceride index (WTI) and modified TyG indices, as useful biomarkers of IR and obesity, are associated with CVD incidence and mortality.^[4]^ However, their associations with all-cause, CVD, and diabetes mellitus (DM) mortality across CKM syndrome stages 0–4 remain unclear. Therefore, using NHANES 1999–2020 data, we examined the associations of TyG, TyG-BMI, WTI and modified TyG indices (triglyceride glucose-waist circumference, TyG-WC; triglyceride glucose multiplied waist-to-height ratio, TyG-WHtR) with all-cause, CVD, and DM mortality in adults with CKM stages 0–4 to inform early risk identification and targeted interventions.

A total of 10,622 adults with CKM stages 0–4 were included (51.14% male). Over a median follow-up of 12.17 years, 2075 participants died, including 625 (5.88%) cardiovascular deaths and 268 (2.52%) DM deaths (Supplementary Figure S1). Higher TyG quartiles were associated with older age, male, worse metabolic profiles, and significantly increased prevalence of metabolic syndrome (MetS), hypertension, diabetes, CVD, CKD (G2-G4), and advanced CKM stages, indicating higher cardiometabolic burden (Supplementary Table S1). Kaplan-Meier analysis demonstrated higher all-cause mortality for TyG above the median, whereas TyG-BMI, WTI, and BMI were not significantly associated with survival (Supplementary Figure S2). After multivariable adjustment (Model III), TyG remained independently associated with all-cause (HR: 1.12, 95% CI: 1.01–1.24) and DM mortality (HR: 1.72, 95% CI: 1.32–2.25), but not with CVD mortality. In contrast, both TyG–BMI and WTI lost significance for all-cause and CVD mortality after full adjustment (Supplementary Table S2). Additive Cox models revealed a J-shaped relationship between TyG and all-cause (inflection point: 9.36) and CVD mortality (inflection point: 9.55), and a nonlinear rise in DM mortality beyond 9.12 (*P* < 0.001). In comparison, TyG–BMI demonstrated U-shaped trends with all-cause and CVD mortality, while WTI showed no significant nonlinear associations (Supplementary Figure S3). Stratified analyses showed significant associations between TyG and all-cause, CVD, and DM mortality in participants < 60 years, and significant associations between TyG and DM mortality in those ≥ 60 years, and elevated TyG predicted higher mortality in male, hypertensive, and diabetic subgroups. In females, TyG was associated with CVD and DM mortality, but not in non-diabetic individuals (Supplementary Figure S4 and S5). These findings highlight the superior and reliable value of the TyG index in high-risk subgroups.

As valuable measures of IR, the TyG index and TyG-BMI have been linked to increased all-cause and CVD mortality across diverse populations. Compared with previous studies, our study has several novel findings. First, our study extends prior evidence by showing that the TyG index predicts all-cause and DM mortality among patients with CKM syndrome (stages 0–4). In contrast to prior evidence linking TyG to CVD mortality, our study revealed no statistically significant relationship in all staged CKM patients. Stratified analyses identified a significant relationship with CVD mortality among hypertensive DM women under 60 years of age (*P* < 0.05). Combined with studies showing no association,^[5]^ our results indicate that TyG may have limited universal predictive value for CVD mortality but could aid risk stratification in specific high-risk subgroups. Furthermore, our study demonstrated that the TyG index outperformed other indices in predicting all-cause, CVD, and DM mortality, particularly among individuals aged < 60 years with both hypertension and diabetes (all *P* < 0.05), highlighting its prognostic value in populations with concurrent cardiometabolic comorbidities. While a previous Chinese cohort reported a significant link between TyG-BMI and higher CVD risk in CKM (stages 0–3),^[4]^ we found no notable connection between TyG-BMI and CVD mortality across CKM (stages 0–4). This discrepancy may stem from including end-stage CKM patients, in whom fluid retention, sarcopenia, malnutrition, and abnormal visceral fat accumulation weaken BMI as a metabolic proxy.^[6]^ Moreover, renal dysfunction and uremic toxins may exacerbate IR and oxidative stress, potentially increasing mortality risk.

To assess whether combining TyG with multiple indices improves mortality prediction, we evaluated TyG-WC and TyG-WHtR for three mortality outcomes and compared five indices for cause-specific CVD mortality. The results are as follows: (1) TyG-WC and TyG-WHtR were not significantly associated with CVD mortality (all *P* > 0.05), whereas TyG-WC was positively correlated with DM mortality and TyG-WHtR with both all-cause and DM mortality, indicating predictive value for diabetes-related mortality (Supplementary Table S3); (2) For cause-specific CVD mortality, TyG, TyG-BMI, and WTI showed no significant associations with cerebrovascular, heart disease, or hypertension mortality (all *P* > 0.05). TyG-WC and TyG-WHtR were also not associated with cerebrovascular mortality (all *P* > 0.05), but both were positively correlated with heart disease mortality (HR: 1.01, 95% CI: 1.00–1.02; HR: 1.23, 95% CI: 1.06–1.42) and hypertension mortality (HR: 1.02, 95% CI: 1.00–1.03; HR: 1.32, 95% CI: 1.08–1.62) (Supplementary Table S4 and Supplementary Figure S6); (3) Additive Cox model revealed J-shaped associations for TyG–WC and TyG-WHtR with heart-disease (inflection point: 109.52 and 6.16) and hypertension mortality (inflection point: 91.70 and 6.44), with risk increasing sharply beyond these thresholds (Supplementary Figure S7); and (4) In stratified analysis, TyG-WC and TyG-WHtR were significantly associated with all-cause, CVD, and DM mortality in individuals < 60 years, but mainly with all-cause mortality in those ≥ 60 years, showing protective patterns and weaker effects than TyG. Associations with CVD mortality were generally nonsignificant across gender, hypertension and diabetes (all *P* > 0.05) (Supplementary Figure S8). These findings reinforce TyG as a strong mortality predictor in CKM stages 0–4, particularly for all-cause, DM, and CVD mortality among hypertensive and diabetic individuals aged < 60 years, whereas TyG-WC and TyG-WHtR better predicted heart disease and hypertension mortality.

**Figure 1:**
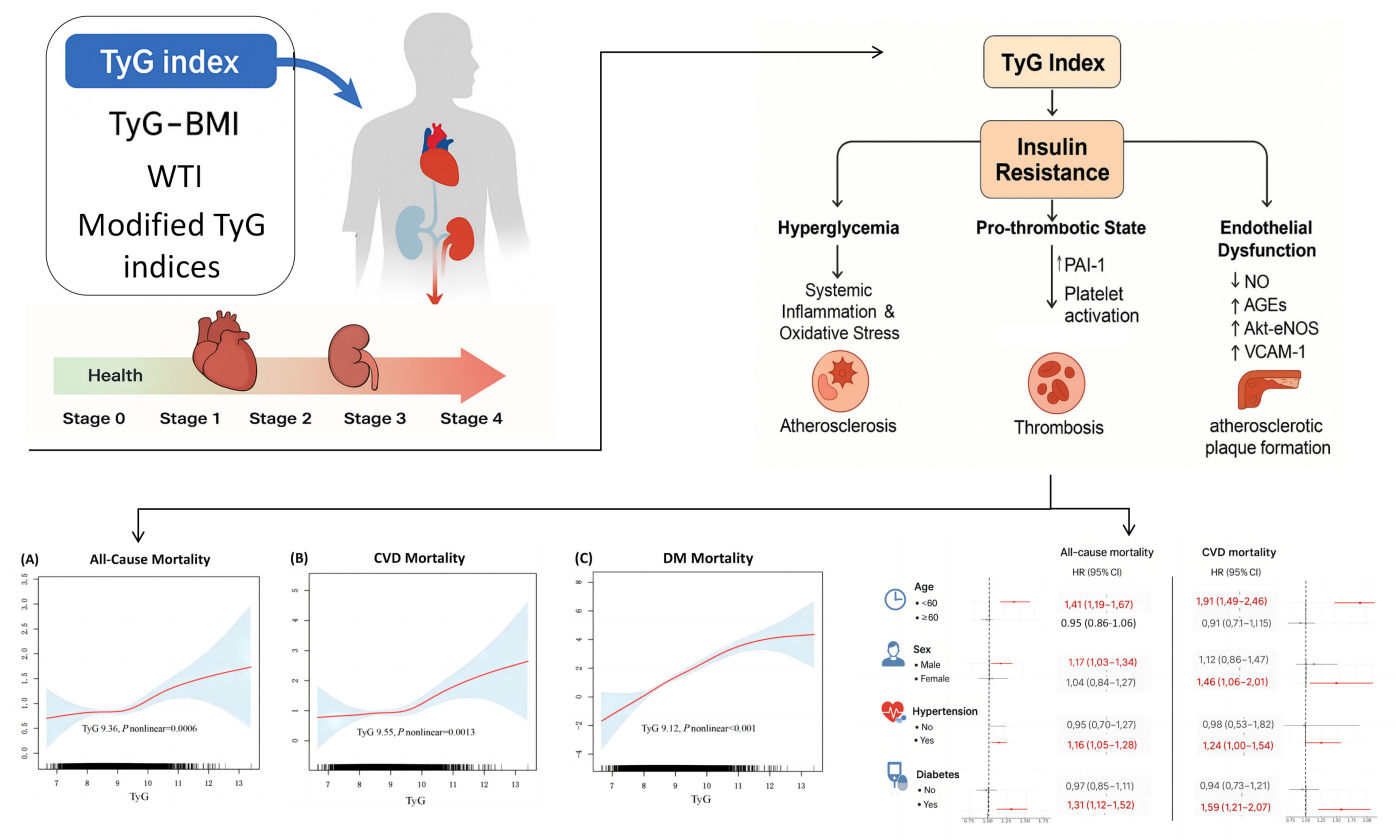
Associations between TyG index and all-cause, CVD, and DM mortality across CKM syndrome stages 0–4. Panels A-C show smooth curve fittings of TyG index with all-cause (A), CVD (B), and DM (C) mortality. The bottom-right forest plots present hazard ratios (HRs) and 95% confidence intervals (CIs) for mortality across subgroups stratified by age, gender, hypertension, and diabetes status.

In this study, while TyG, TyG-BMI, and WTI showed no significant association with CVD mortality, TyG was significantly associated with CVD mortality in specific high-risk populations (Figure 1). This may be related to the complex pathophysiology of CKM end-stage patients and the multifactorial nature of CVD mortality. As markers of IR and metabolic syndrome risk, TyG and TyG-BMI may show heterogeneous associations across CVD subtypes. Although TyG was not significantly associated with CVD mortality, persistently elevated TyG was closely linked to higher risks of incident CVD and DM, potentially increasing all-cause, CVD, and DM mortality in CKM syndrome patients. Previous studies have suggested that combining TyG with obesity-related indices enhances its correlation with disease outcomes and improves diagnostic performance.^[7,8]^ For example, TyG-WC has been linked to all-cause mortality in CKM stages 0–3, and TyG-WHtR to CVD mortality.^[9]^ In contrast, our study observed no significant associations of TyG-WC or TyG-WHtR with all-cause, CVD, or DM mortality, although both were significantly associated with heart disease and hypertension mortality. This discrepancy may reflect the complex end-stage CKM milieu, confounding by non-metabolic factors and the multifactorial nature of CVD mortality. Overall, TyG may predict CVD mortality in specific high-risk populations, whereas TyG-WC and TyG-WHtR may better predict heart disease and hypertension mortality.

Although the precise mechanisms by which the TyG index contributes to increased mortality are unknown, IR may play a critical role in this relationship. IR manifests as diminished responsiveness to insulin in skeletal muscle and target tissues, resulting in decreased glucose uptake and hyperglycemia, which may drive CVD pathogenesis and accelerate CKM syndrome progression through multiple pathways. A hyperglycemia-induced imbalance in glucose metabolism triggers systemic inflammation and oxidative stress, accelerating atherosclerosis progression.^[10]^ IR promotes thrombosis by inducing fibrinogen activator inhibitor-1 production and enhancing platelet activation *via* increased levels of thromboxane A2-responsive tissue factors and platelet-derived molecules involved in cell adhesion.^[11]^ IR also impairs endothelial function through multiple mechanisms, including nitric oxide (NO) inactivation and advanced glycation end products (AGEs) buildup,^[12]^ as well as the suppression of the insulin receptor-Akt-eNOS signaling pathway and the increased expression of vascular cell adhesion molecule-1 (VCAM-1), collectively accelerating atherosclerotic plaque formation.^[13]^ Additionally, IR has been implicated in vascular smooth muscle cell proliferation and cardiac fibrosis, both of which significantly contribute to heart failure risk.

In conclusion, compared with TyG–BMI and WTI, the TyG index was more strongly associated with all-cause, CVD, and DM mortality among U.S. adults with CKM syndrome stages 0-4 and showed superior predictive efficacy for mortality risk assessment. Although TyG–WC and TyG–WHtR were associated with heart disease and hypertension mortality, TyG showed the strongest overall associations and the best overall predictive performance. These findings underscore the clinical value of monitoring TyG in CKM stages 0-4, particularly in adults aged <60 years. However, residual confounding may remain despite extensive covariate adjustment, and generalizability may be limited to U.S. adults given ethnic and geographic differences in metabolic phenotypes. Future studies should validate these results prospectively in diverse populations and CKM stages and clarify the biological mechanisms linking TyG to organ dysfunction. Integrating TyG into clinical risk models may improve early identification and support tailored interventions in CKM syndrome, ultimately enhancing patient outcomes.

## Supplementary Material

Supplementary Material Details
