# Supplementary material for: Associations of the triglyceride-glucose index, triglyceride glucose-body mass index, waist-triglyceride index and modified triglyceride-glucose indices with mortality in cardiovascular-kidney-metabolic syndrome stages 0–4: Evidence from NHANES 1999–2020
Source: J Transl Int Med. 2026 Feb 13;14(1):158–61. doi: 10.1515/jtim-2026-0014 (PMC12916274; doi:10.1515/jtim-2026-0014)
Supplement: Supplementary file 1 — Supplementary Material Details [file jtim-2026-0014_sm.pdf]

Supplementary materials

Associations of the triglyceride-glucose index, triglyceride glucose-body mass index, waist-triglyceride index and modified triglyceride-glucose indices with mortality in cardiovascular-kidney-metabolic syndrome stages 0–4: evidence from NHANES 1999–2020

Jingya Zhao[1,2], Xinning Lu[1], Hui Wang[1], Qin Chen[2], Yigang Wan[1,3,4]

[1]Department of Traditional Chinese Medicine, Nanjing Drum Tower Hospital Clinical College of Nanjing University of Chinese Medicine, Nanjing 211166, Jiangsu Province, China

[2]Department of Nephrology, Tongde Hospital of Zhejiang Province, Hangzhou 310012, Zhejiang Province, China

[3]Department of Traditional Chinese Medicine, Nanjing Drum Tower Hospital, Affiliated Hospital of Medical School, Nanjing University, Nanjing 211166, Jiangsu Province, China

[4]Institute of Chinese Medicine, Nanjing University, Nanjing 211166, Jiangsu Province, China

Address for Correspondence:

Yigang Wan, Department of Traditional Chinese Medicine, Nanjing Drum Tower Hospital Clinical College of Nanjing University of Chinese Medicine, No.321 Zhongshan Road, Nanjing 210008, Jiangsu Province, China. Email: wyg68918@sina.com. https://orcid.org/0000-0002-5278-2695

Qin Chen, Department of Nephrology, Tongde Hospital of Zhejiang Province, No.234 Guicui Road, Hangzhou 310012, Zhejiang Province, China. Email: chenqin2858@163.com. https://orcid.org/0000-0001-8740-0354

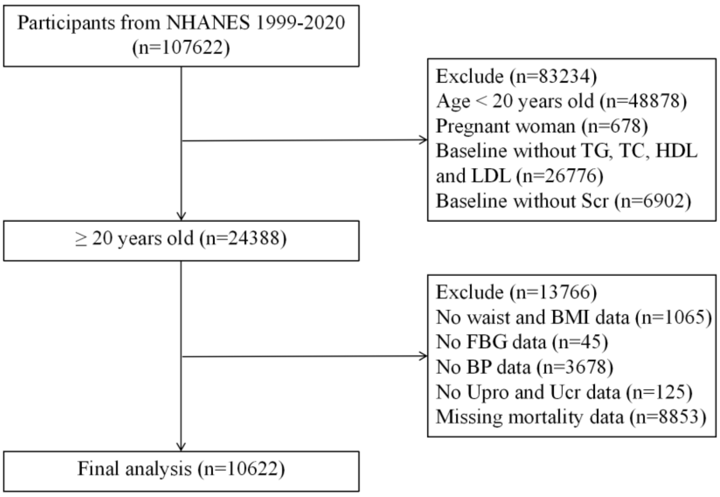

Supplementary Figure S1: Flowchart of the study population.

Supplementary Table S1: Baseline characteristics by quartiles of the TyG index in individuals with CKM syndrome stages 0-4, NHANES 1999 to 2020

**Characteristic**	**Quartile 1**	**Quartile 2**	**Quartile 3**	**Quartile 4**	***P* value**
N (%)	2618 (24.65%)	2684 (25.27%)	2616 (24.63%)	2704 (25.45%)	
Age, years	41.82 ± 16.58	49.48 ± 18.02	52.52 ± 17.55	55.06 ± 16.06	<0.001
Sex, *n* (%)					<0.001
Male	1075 (41.06%)	1358 (50.60%)	1441 (55.08%)	1558 (57.62%)	
Female	1543 (58.94%)	1326 (49.40%)	1175 (44.92%)	1146 (42.38%)	
Race, *n* (%)					<0.001
Mexican American	366 (13.98%)	487 (18.14%)	560 (21.41%)	700 (25.89%)	
Other Hispanic	172 (6.57%)	188 (7.00%)	215 (8.22%)	222 (8.21%)	

Non-Hispanic White	1216 (46.45%)	1339 (49.89%)	1361 (52.03%)	1365 (50.48%)	
Non-Hispanic Black	747 (28.53%)	539 (20.08%)	346 (13.23%)	305 (11.28%)	
Other Races	117 (4.47%)	131 (4.88%)	134 (5.12%)	112 (4.14%)	
Educational levels, *n* (%)					<0.001
Less than 9th grade	192 (7.33%)	320 (11.92%)	409 (15.63%)	540 (19.97%)	
9–11th grade	374 (14.29%)	402 (14.98%)	412 (15.75%)	471 (17.42%)	
High school graduate	531 (20.28%)	681 (25.37%)	620 (23.70%)	675 (24.96%)	
Some college or AA degree	840 (32.09%)	745 (27.76%)	660 (25.23%)	654 (24.19%)	
College graduate or above	679 (25.94%)	536 (19.97%)	511 (19.53%)	358 (13.24%)	
PIR, *n* (%)					<0.001
≥3	1107 (42.28%)	1050 (39.12%)	986 (37.69%)	897 (33.17%)	
1-3	1095 (41.83%)	1182 (44.04%)	1202 (45.95%)	1327 (49.08%)	
<1	416 (15.89%)	452 (16.84%)	428 (16.36%)	480 (17.75%)	
Smoking status, *n* (%)					<0.001
No	1603 (61.23%)	1408 (52.46%)	1304 (49.85%)	1215 (44.93%)	
Yes	1015 (38.77%)	1276 (47.54%)	1312 (50.15%)	1489 (55.07%)	
Alcohol consumption, *n* (%)					<0.001
Never	676 (25.82%)	751 (27.98%)	829 (31.69%)	1004 (37.13%)	

Mild	1936 (73.95%)	1923 (71.65%)	1781 (68.08%)	1695 (62.68%)	
Moderate	1 (0.04%)	5 (0.19%)	3 (0.11%)	0 (0.00%)	
Heavy	5 (0.19%)	5 (0.19%)	3 (0.11%)	5 (0.18%)	
SBP (mmHg)	117.16 ± 17.17	123.11 ± 18.36	125.70 ± 18.59	129.11 ± 19.05	<0.001
DBP (mmHg)	68.07 ± 11.93	69.56 ± 11.97	70.80 ± 12.68	71.79 ± 12.79	<0.001
BMI (kg/m^2)	26.15 ± 5.99	27.94 ± 5.92	29.23 ± 5.92	30.59 ± 5.86	<0.001
WC (cm)	89.73 ± 14.39	96.26 ± 14.32	100.39 ± 14.15	104.98 ± 13.86	<0.001
BUN (mg/dL)	12.43 ± 4.48	13.03 ± 5.24	13.76 ± 6.26	14.30 ± 6.09	<0.001
BUA (mg/dL)	4.92 ± 1.27	5.41 ± 1.33	5.74 ± 1.36	5.94 ± 1.46	<0.001
SCr (mg/dL)	0.83 ± 0.24	0.87 ± 0.32	0.89 ± 0.37	0.91 ± 0.43	<0.001
eGFR (mL/min/1.73m^2)	101.98 ± 20.25	96.16 ± 21.10	94.06 ± 22.03	91.76 ± 22.61	<0.001
FBG (mg/dL)	92.55 ± 10.20	98.99 ± 13.30	104.66 ± 19.08	132.21 ± 57.72	<0.001
HbA1c (%)	5.30 ± 0.43	5.46 ± 0.55	5.60 ± 0.70	6.31 ± 1.64	<0.001
HDL-c (mg/dL)	61.67 ± 16.46	56.10 ± 14.96	50.30 ± 13.14	43.69 ± 11.68	<0.001
LDL-c (mg/dL)	105.43 ± 30.07	118.79 ± 33.37	124.90 ± 35.64	121.77 ± 36.19	<0.001
TC (mg/dL)	179.34 ± 35.21	194.24 ± 36.98	203.17 ± 39.55	215.44 ± 47.32	<0.001
TG (mg/dL)	62.09 ± 14.22	97.17 ± 15.49	138.81 ± 24.85	262.91 ± 192.02	<0.001
Diabetes, *n* (%)	83 (3.17%)	200 (7.45%)	366 (13.99%)	1008 (37.28%)	<0.001
MetS, *n* (%)	511 (19.52%)	1024 (38.15%)	1541 (58.91%)	2215 (81.92%)	<0.001

Hypertension, *n* (%)	873 (33.35%)	1339 (49.89%)	1499 (57.30%)	1797 (66.46%)	<0.001
CVD, *n* (%)	122 (4.66%)	209 (7.79%)	264 (10.09%)	390 (14.42%)	<0.001
FRS levels, *n* (%)					<0.001
Low	2143 (81.86%)	1694 (63.11%)	1356 (51.83%)	1051 (38.87%)	
Moderate	238 (9.09%)	473 (17.62%)	591 (22.59%)	652 (24.11%)	
High	237 (9.05%)	517 (19.26%)	669 (25.57%)	1001 (37.02%)	
CKD stage, *n* (%)					<0.001
G1	1932 (73.80%)	1755 (65.39%)	1637 (62.58%)	1609 (59.50%)	
G2	597 (22.80%)	775 (28.87%)	772 (29.51%)	843 (31.18%)	
G3	83 (3.17%)	140 (5.22%)	189 (7.22%)	219 (8.10%)	
G4	5 (0.19%)	10 (0.37%)	11 (0.42%)	27 (1.00%)	
G5	1 (0.04%)	4 (0.15%)	7 (0.27%)	6 (0.22%)	
CKM syndrome stage, *n* (%)					<0.001
0	747 (28.53%)	309 (11.51%)	36 (1.38%)	0 (0.00%)	
1	810 (30.94%)	775 (28.87%)	228 (8.72%)	14 (0.52%)	
2	755 (28.84%)	968 (36.07%)	1553 (59.37%)	1525 (56.40%)	
3	180 (6.88%)	417 (15.54%)	533 (20.37%)	765 (28.29%)	
4	127 (4.81%)	218 (8.01%)	277 (10.16%)	420 (14.79%)	
TyG	7.93 ± 0.25	8.46 ± 0.12	8.86 ± 0.12	9.58 ± 0.50	<0.001
TyG–BMI	207.48 ± 49.02	236.37 ± 50.43	259.02 ± 52.80	293.16 ± 58.66	<0.001
TyG–WC	711.96 ±	814.23 ±	889.66 ±	1006.32 ±	<0.001

	120.65	122.51	126.77	145.75	
TyG–WHtR	4.25 ± 0.73	4.85 ± 0.73	5.31 ± 0.75	6.00 ± 0.87	<0.001
WTI	63.05 ± 18.17	105.35 ± 22.02	156.86 ± 33.81	310.77 ± 231.44	<0.001
All-cause mortality, *n* (%)	271 (10.35%)	495 (18.44%)	566 (21.64%)	743 (27.48%)	<0.001
CVD mortality, *n* (%)	71 (2.71%)	159 (5.92%)	169 (6.46%)	226 (8.36%)	<0.001
DM mortality, *n* (%)	9 (0.34%)	31 (1.15%)	62 (2.37%)	166 (6.14%)	<0.001

SBP: systolic blood pressure; DBP: diastolic blood pressure; BMI: body mass index; WC: waist circumference; BUN: blood urea nitrogen; BUA: blood uric acid; SCr: serum creatinine; eGFR: estimation of glomerular filtration rate; FBG: fasting blood glucose; HbA1c: hemoglobin A1c; HDL-C: high-density lipoprotein cholesterol; LDL-C: low-density lipoprotein cholesterol; TC: total cholesterol; TG: total triglyceride; PIR: family poverty–income ratio; MetS: metabolic syndrome; FRS: framingham coronary heart disease risk score; CKD: chronic kidney disease; CKM: cardiovascular–kidney–metabolic; TyG: triglyceride–glucose; TyG–BMI: triglyceride glucose–body mass index; TyG–WC: triglyceride glucose–waist circumference; TyG–WHtR: triglyceride glucose multiplied waist-to-height ratio; WTI: waist–triglyceride index; CVD: cardiovascular disease; DM: diabetes mellitus.

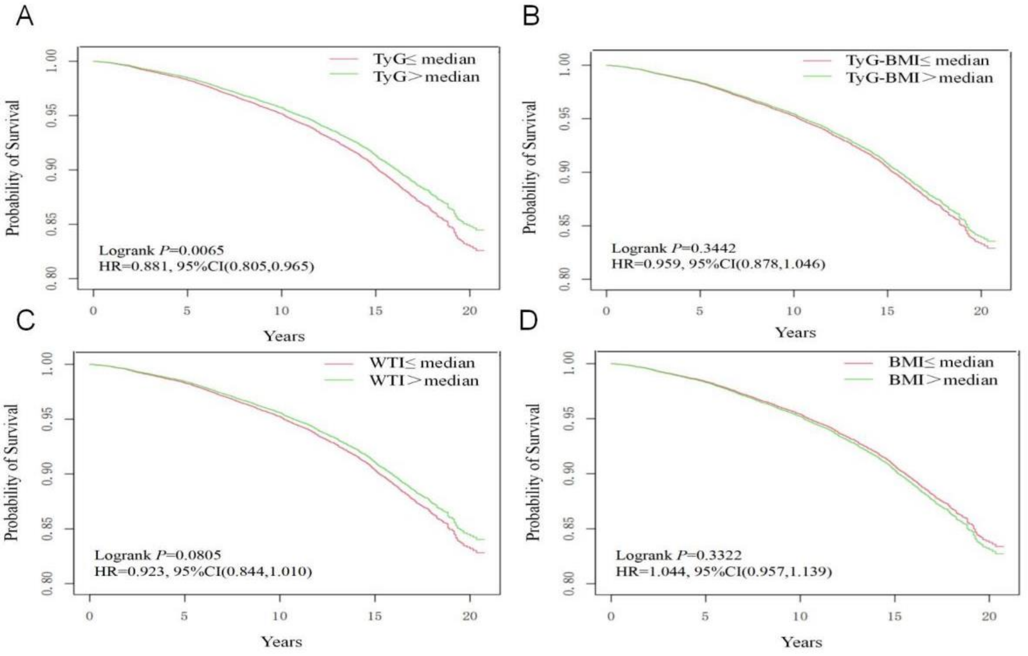

Supplementary Figure S2: Associations of the TyG index (A), TyG–BMI (B), WTI (C) and BMI (D) with all-cause mortality in patients with CKM syndrome stages 0-4, analyzed through Kaplan–Meier analysis.

Supplementary Table S2: Multivariable Cox regression analyses of the TyG index, TyG–BMI and WTI with all-cause, CVD and DM mortality in patients with CKM syndrome stages 0-4

	**Model I HR（95% CI)**	***P* value**	**Model II HR（95% CI)**	***P* value**	**Model III HR（95% CI)**	***P* value**
All-cause mortality						
TyG (continuous)	1.69 (1.56, 1.82)	<0.0001	1.31 (1.21, 1.43)	<0.0001	1.12 (1.01, 1.24)	0.0374
Quartile 1	Ref		Ref		Ref	
Quartile 2	1.79 (1.42, 2.26)	<0.0001	1.00 (0.79, 1.26)	0.9919	1.00 (0.79, 1.25)	0.9689
Quartile 3	2.22 (1.79, 2.75)	<0.0001	1.03 (0.84, 1.28)	0.7530	0.96 (0.76, 1.20)	0.6974
Quartile 4	3.04 (2.46, 3.77)	<0.0001	1.33 (1.10, 1.61)	0.0029	1.01 (0.81, 1.25)	0.9226
TyG–BMI10 (continuous)	1.03 (1.02, 1.04)	<0.0001	1.02 (1.01, 1.02)	<0.0001	1.00 (0.99, 1.01)	0.9901
Quartile 1	Ref		Ref		Ref	
Quartile 2	1.31 (1.10, 1.56)	0.0023	0.84 (0.73, 0.98)	0.0218	0.78 (0.67, 0.91)	0.0018
Quartile 3	1.46 (1.24, 1.72)	<0.0001	0.89 (0.77, 1.02)	0.0933	0.77 (0.65, 0.91)	0.0017
Quartile 4	1.61 (1.37, 1.90)	<0.0001	1.15 (0.99, 1.34)	0.0626	0.82 (0.68, 0.99)	0.0358
WTI10 (continuous)	1.01 (1.00, 1.01)	<0.0001	1.00 (1.00, 1.01)	0.0085	1.00 (0.99, 1.00)	0.5104
Quartile 1	Ref		Ref		Ref	

Quartile 2	1.90 (1.57, 2.31)	<0.0001	1.05 (0.86, 1.27)	0.6504	1.01 (0.83, 1.23)	0.9307
Quartile 3	2.11 (1.77, 2.52)	<0.0001	1.06 (0.89, 1.26)	0.5423	0.94 (0.78, 1.13)	0.5281
Quartile 4	2.63 (2.18, 3.17)	<0.0001	1.31 (1.11, 1.55)	0.0014	1.05 (0.87, 1.28)	0.5854
CVD mortality						
TyG (continuous)	1.76 (1.58, 1.97)	<0.0001	1.42 (1.22, 1.64)	<0.0001	1.21 (0.99, 1.48)	0.0619
Quartile 1	Ref		Ref		Ref	
Quartile 2	2.51 (1.76, 3.58)	<0.0001	1.31 (0.92, 1.87)	0.1363	1.31 (0.91, 1.90)	0.1444
Quartile 3	3.19 (2.26, 4.51)	<0.0001	1.40 (0.99, 1.99)	0.0567	1.32 (0.88, 1.99)	0.1855
Quartile 4	4.08 (2.93, 5.67)	<0.0001	1.71 (1.24, 2.37)	0.0011	1.33 (0.88, 1.99)	0.1730
TyG–BMI10 (continuous)	1.04 (1.02, 1.05)	<0.0001	1.04 (1.02, 1.05)	<0.0001	1.01 (0.99, 1.03)	0.3909
Quartile 1	Ref		Ref		Ref	
Quartile 2	1.47 (1.09, 1.99)	0.0127	0.93 (0.69, 1.23)	0.5956	0.85 (0.63, 1.14)	0.2707
Quartile 3	1.52 (1.17, 1.98)	0.0019	0.91 (0.70, 1.18)	0.4782	0.75 (0.55, 1.04)	0.0841
Quartile 4	1.97 (1.50, 2.57)	<0.0001	1.45 (1.12, 1.88)	0.0044	0.95 (0.68, 1.31)	0.7380
WTI10 (continuous)	1.01 (1.00, 1.01)	<0.0001	1.00 (1.00, 1.01)	0.0066	1.00 (0.99, 1.01)	0.2768
Quartile 1	Ref		Ref		Ref	
Quartile 2	2.72 (2.01, 3.67)	<0.0001	1.39 (1.03, 1.87)	0.0329	1.39 (1.02, 1.90)	0.0362
Quartile 3	2.81 (2.05, 3.84)	<0.0001	1.33 (0.98, 1.80)	0.0640	1.20 (0.85, 1.71)	0.3017

Quartile 4	3.35 (2.42, 4.64)	<0.0001	1.65 (1.21, 2.26)	0.0017	1.36 (0.94, 1.97)	0.1018
DM mortality						
TyG (continuous)	3.06 (2.54, 3.69)	<0.0001	3.09 (2.51, 3.81)	<0.0001	1.72 (1.32, 2.25)	<0.0001
Quartile 1	Ref		Ref		Ref	
Quartile 2	2.45 (0.98, 6.15)	0.0561	1.45 (0.58, 3.63)	0.4293	1.14 (0.44, 2.93)	0.7925
Quartile 3	4.71 (1.96, 11.33)	0.0005	2.35 (0.96, 5.77)	0.0610	1.22 (0.44, 3.36)	0.7002
Quartile 4	16.02 (6.96, 36.90)	<0.0001	7.67 (3.30, 17.83)	<0.0001	1.89 (0.66, 5.39)	0.2332
TyG–BMI10 (continuous)	1.10 (1.08, 1.12)	<0.0001	1.12 (1.09, 1.14)	<0.0001	1.04 (1.00, 1.07)	0.0310
Quartile 1	Ref		Ref		Ref	
Quartile 2	3.07 (1.55, 6.08)	0.0013	1.97 (1.00, 3.88)	0.0497	1.03 (0.51, 2.08)	0.9284
Quartile 3	5.06 (2.64, 9.71)	<0.0001	3.12 (1.60, 6.09)	0.0009	1.16 (0.56, 2.39)	0.6958
Quartile 4	8.78 (4.85, 15.87)	<0.0001	6.21 (3.42, 11.29)	<0.0001	1.16 (0.55, 2.49)	0.6937
WTI10 (continuous)	1.01 (1.01, 1.01)	<0.0001	1.01 (1.00, 1.01)	<0.0001	1.01 (1.00, 1.01)	0.0030
Quartile 1	Ref		Ref		Ref	
Quartile 2	2.09 (0.94, 4.64)	0.0703	1.17 (0.53, 2.61)	0.6976	0.84 (0.38, 1.84)	0.6632
Quartile 3	2.99 (1.50, 5.98)	0.0019	1.56 (0.76, 3.19)	0.2262	0.74 (0.33, 1.65)	0.4673
Quartile 4	7.83 (3.79, 16.19)	<0.0001	4.26 (2.01, 9.04)	0.0002	1.34 (0.52, 3.43)	0.5461

Model I: No adjustment; Model II: Adjusted for age, sex, and race; Model III: Adjusted for age, sex, race, educational level, PIR, BUN, BUA, SCr, HDL-C, LDL-C, smoking status, alcohol

consumption, hypertension and diabetes. TyG–BMI10: triglyceride glucose–body mass index divided by 10; WTI10: waist-triglyceride index divided by 10.

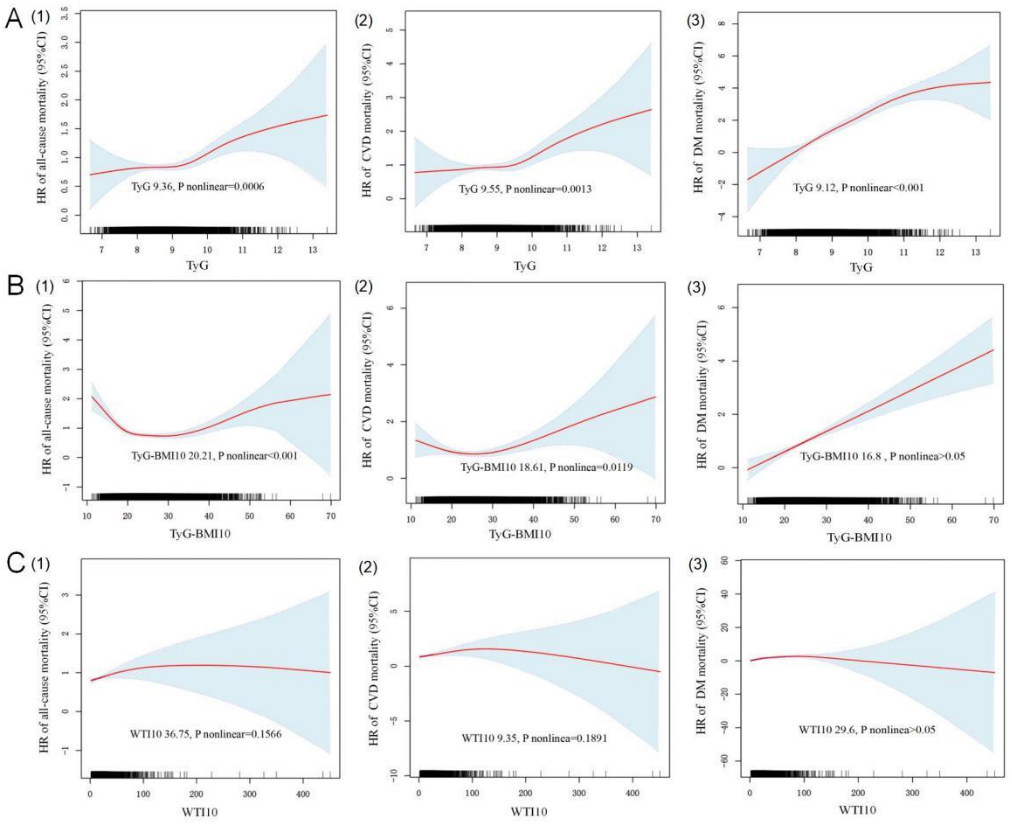

Supplementary Figure S3: Smooth curve fitting of the TyG index (A), TyG–BMI (B) and WTI (C) with all-cause, CVD and DM mortality in patients with CKM syndrome stages 0-4.

Variable(s)	All-cause mortality HR（95%CI）	*P* value	CVD mortality HR（95%CI）	*P* value	DM mortality HR（95%CI）	*P* value
<60						
TyG	1.41 (1.19, 1.67)	<0.0001	1.91 (1.49, 2.46)	<0.0001	1.92 (1.45, 2.54)	<0.0001
TyG-BMI10	1.02 (1.00, 1.05)	0.1049	1.03 (0.99, 1.07)	0.2029	1.05 (1.02, 1.09)	0.0041
WTI10	1.00 (1.00, 1.01)	0.1620	1.01 (1.00, 1.01)	0.0081	1.00 (1.00, 1.01)	0.1639
≥60						
TyG	0.95 (0.86, 1.06)	0.3437	0.91 (0.71, 1.15)	0.4123	1.51 (1.19, 1.90)	0.0005
TyG-BMI10	0.96 (0.95, 0.97)	<0.0001	0.97 (0.94, 0.99)	0.0358	0.98 (0.95, 1.01)	0.2022
WTI10	0.99 (0.98, 1.00)	0.0087	0.99 (0.98, 1.00)	0.1222	1.00 (1.00, 1.01)	0.1272
Male						
TyG	1.17 (1.03, 1.34)	0.0196	1.12 (0.86, 1.47)	0.3942	1.69 (1.22, 2.33)	0.0014
TyG-BMI10	1.02 (0.99, 1.04)	0.0730	1.02 (0.98, 1.06)	0.2559	1.05 (1.01, 1.08)	0.0135
WTI10	1.00 (0.99, 1.00)	0.2241	1.00 (0.99, 1.01)	0.3905	1.00 (1.00, 1.01)	0.0210
Female						
TyG	1.04 (0.84, 1.27)	0.7458	1.46 (1.06, 2.01)	0.0200	1.72 (1.05, 2.81)	0.0323
TyG-BMI10	0.98 (0.97, 1.00)	0.0650	1.00 (0.97, 1.03)	0.7938	1.03 (0.97, 1.09)	0.3421
WTI10	1.00 (0.99, 1.01)	0.4115	1.00 (0.99, 1.02)	0.5794	1.01 (0.99, 1.03)	0.2487

Supplementary Figure S4: Stratified analyses of the TyG index, TyG–BMI and WTI with all-cause, CVD and DM mortality across age and gender in patients with CKM syndrome stages 0-4.

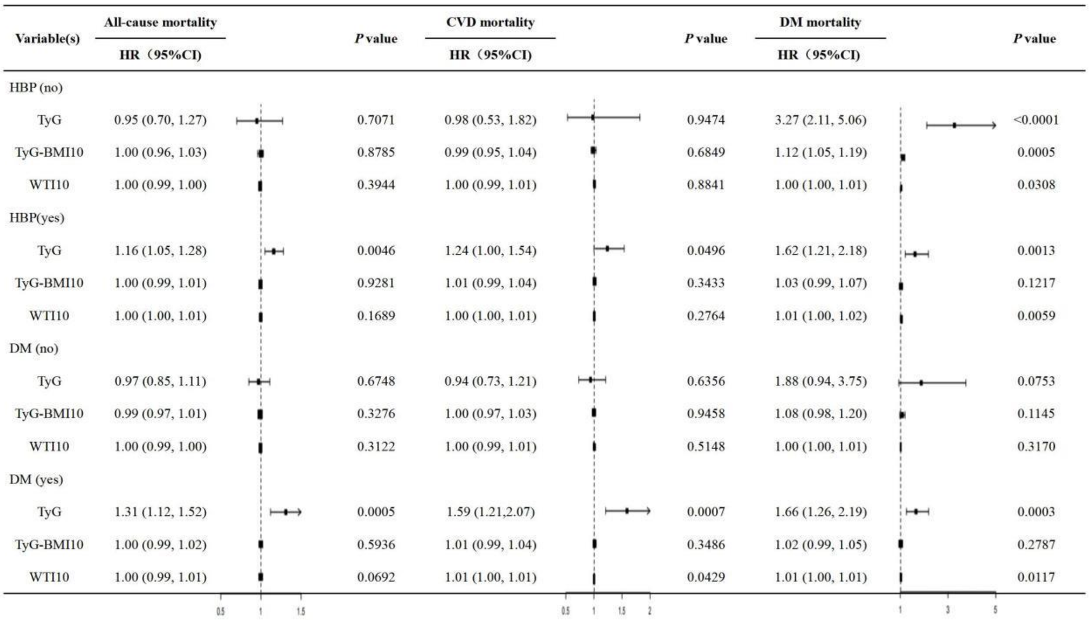

Variable(s)	All-cause mortality HR（95%CI)	*P* value	CVD mortality HR（95%CI)	*P* value	DM mortality HR（95%CI)	*P* value
HBP (no)						
TyG	0.95 (0.70, 1.27)	0.7071	0.98 (0.53, 1.82)	0.9474	3.27 (2.11, 5.06)	<0.0001
TyG-BMI10	1.00 (0.96, 1.03)	0.8785	0.99 (0.95, 1.04)	0.6849	1.12 (1.05, 1.19)	0.0005
WTI10	1.00 (0.99, 1.00)	0.3944	1.00 (0.99, 1.01)	0.8841	1.00 (1.00, 1.01)	0.0308
HBP(yes)						
TyG	1.16 (1.05, 1.28)	0.0046	1.24 (1.00, 1.54)	0.0496	1.62 (1.21, 2.18)	0.0013
TyG-BMI10	1.00 (0.99, 1.01)	0.9281	1.01 (0.99, 1.04)	0.3433	1.03 (0.99, 1.07)	0.1217
WTI10	1.00 (1.00, 1.01)	0.1689	1.00 (1.00, 1.01)	0.2764	1.01 (1.00, 1.02)	0.0059
DM (no)						
TyG	0.97 (0.85, 1.11)	0.6748	0.94 (0.73, 1.21)	0.6356	1.88 (0.94, 3.75)	0.0753
TyG-BMI10	0.99 (0.97, 1.01)	0.3276	1.00 (0.97, 1.03)	0.9458	1.08 (0.98, 1.20)	0.1145
WTI10	1.00 (0.99, 1.00)	0.3122	1.00 (0.99, 1.01)	0.5148	1.00 (1.00, 1.01)	0.3170
DM (yes)						
TyG	1.31 (1.12, 1.52)	0.0005	1.59 (1.21,2.07)	0.0007	1.66 (1.26, 2.19)	0.0003
TyG-BMI10	1.00 (0.99, 1.02)	0.5936	1.01 (0.99, 1.04)	0.3486	1.02 (0.99, 1.05)	0.2787
WTI10	1.00 (0.99, 1.01)	0.0692	1.01 (1.00, 1.01)	0.0429	1.01 (1.00, 1.01)	0.0117

Supplementary Figure S5: Stratified analyses of the TyG index, TyG–BMI and WTI with all-cause, CVD and DM mortality across HBP and DM in patients with CKM syndrome stages 0-4.

Supplementary Table S3: Multivariable Cox regression analyses of the modified TyG indices with all-cause, CVD and DM mortality in patients with CKM syndrome stages 0-4

	Model I HR（95% CI)	*P* value	Model II HR（95% CI)	*P* value	Model III HR（95% CI)	*P* value
All-cause mortality						
TyG-WC10 (continuous)	1.02 (1.02, 1.03)	<0.0001	1.01 (1.01, 1.01)	<0.0001	1.00 (0.99, 1.01)	0.0959
Quartile 1	Ref		Ref		Ref	
Quartile 2	1.78 (1.46, 2.18)	<0.0001	0.96 (0.80, 1.15)	0.6465	0.92 (0.77, 1.11)	0.4052
Quartile 3	2.11 (1.76,	<0.0001	0.91 (0.76,	0.2809	0.78 (0.64,	0.0085

	2.53)		1.08)		0.94)	
Quartile 4	3.06 (2.55, 3.67)	<0.0001	1.31 (1.11, 1.55)	0.0013	0.94 (0.77, 1.13)	0.4853
TyG–WHtR(continuous)	1.55 (1.47, 1.63)	<0.0001	1.22 (1.16, 1.30)	<0.0001	1.08 (1.00, 1.16)	0.0445
Quartile 1	Ref		Ref		Ref	
Quartile 2	1.88 (1.56, 2.27)	<0.0001	0.96 (0.80, 1.14)	0.6246	0.92 (0.76, 1.11)	0.3700
Quartile 3	2.33 (1.89, 2.87)	<0.0001	0.97 (0.80, 1.17)	0.7285	0.85 (0.68, 1.06)	0.1515
Quartile 4	3.74 (3.09, 4.53)	<0.0001	1.39 (1.17, 1.66)	0.0002	1.00 (0.80, 1.24)	0.9791
CVD mortality						
TyG-WC10 (continuous)	1.03 (1.02, 1.03)	<0.0001	1.02 (1.01, 1.02)	<0.0001	1.01 (0.99, 1.02)	0.0909
Quartile 1	Ref		Ref		Ref	
Quartile 2	2.68 (1.86, 3.86)	<0.0001	1.35 (0.95, 1.92)	0.0901	1.29 (0.91, 1.84)	0.1579
Quartile 3	2.77 (2.05, 3.75)	<0.0001	1.11 (0.81, 1.52)	0.5095	0.93 (0.67, 1.30)	0.6821
Quartile 4	4.57 (3.31, 6.31)	<0.0001	1.87 (1.38, 2.52)	<0.0001	1.26 (0.87, 1.82)	0.2162
TyG–WHtR(continuous)	1.65 (1.53, 1.78)	<0.0001	1.34 (1.21, 1.49)	<0.0001	1.16 (0.99, 1.34)	0.0572
Quartile 1	Ref		Ref		Ref	
Quartile 2	2.55 (1.85, 3.52)	<0.0001	1.21 (0.89, 1.65)	0.2256	1.14 (0.81, 1.60)	0.4441
Quartile 3	2.89 (2.05, 4.07)	<0.0001	1.11 (0.80, 1.53)	0.5338	0.93 (0.62, 1.37)	0.6986
Quartile 4	5.04 (3.62, 7.01)	<0.0001	1.76 (1.28, 2.42)	0.0005	1.17 (0.76, 1.82)	0.4798
DM mortality						

TyG-WC10(continuous)	1.05 (1.05, 1.06)	<0.0001	1.06 (1.05, 1.07)	<0.0001	1.02 (1.01, 1.04)	0.0002
Quartile 1	Ref		Ref		Ref	
Quartile 2	4.67 (1.99, 10.96)	0.0004	2.56 (1.09, 5.99)	0.0302	1.63 (0.70, 3.77)	0.2579
Quartile 3	5.67 (2.53, 12.71)	<0.0001	2.60 (1.15, 5.89)	0.0217	0.86 (0.37, 2.00)	0.7305
Quartile 4	23.13 (10.99,48.66)	<0.0001	10.71 (4.99, 22.96)	<0.0001	2.01 (0.83, 4.83)	0.1203
TyG–WHtR(continuous)	2.53 (2.26, 2.82)	<0.0001	2.48 (2.14, 2.86)	<0.0001	1.42 (1.18, 1.72)	0.0002
Quartile 1	Ref		Ref		Ref	
Quartile 2	6.63 (2.64, 16.66)	<0.0001	3.54 (1.42, 8.82)	0.0066	2.19 (0.89, 5.42)	0.0885
Quartile 3	6.79 (2.73, 16.87)	<0.0001	3.02 (1.22, 7.46)	0.0166	1.16 (0.43, 3.18)	0.7671
Quartile 4	32.93 (14.14,76.71)	<0.0001	13.52 (5.77, 31.69)	<0.0001	2.66 (0.98, 7.25)	0.0551

Model I: No adjustment; Model II: Adjusted for age, sex, and race; Model III: Adjusted for age, sex, race, educational level, PIR, BUN, BUA, SCr, HDL-C, LDL-C, smoking status, alcohol consumption, hypertension and diabetes; TyG–WC10: triglyceride glucose–waist circumference divided by 10; TyG–WHtR: triglyceride glucose multiplied waist-to-height ratio.

Supplementary Table S4: Multivariable Cox regression analyses of TyG, TyG-BMI, WTI and modified TyG indices for cause-specific cardiovascular disease mortality in patients with CKM syndrome stages 0-4

	Model I	*P* value	Model II	*P* value	Model III	*P* value

	HR（95% CI)		HR（95% CI)		HR（95% CI)	
Heart diseases						
TyG	1.76 (1.55, 2.00)	<0.0001	1.41 (1.19, 1.68)	<0.0001	1.24 (0.99, 1.53)	0.0545
TyG-BMI10	1.04 (1.03, 1.06)	<0.0001	1.04 (1.02, 1.06)	<0.0001	1.02 (0.99, 1.04)	0.0888
WTI10	1.01 (1.00, 1.01)	<0.0001	1.00 (1.00, 1.01)	0.0068	1.00 (0.99, 1.01)	0.1727
TyG-WC10	1.03 (1.02, 1.03)	<0.0001	1.02 (1.01, 1.03)	<0.0001	1.01 (1.00, 1.02)	0.0095
TyG–WHtR	1.68 (1.55, 1.82)	<0.0001	1.38 (1.24, 1.54)	<0.0001	1.23 (1.06, 1.42)	0.0066
Cerebrovascular diseases						
TyG	1.79 (1.43, 2.24)	<0.0001	1.43 (0.99, 2.07)	0.0587	1.10 (0.67, 1.81)	0.7152
TyG-BMI10	1.01 (0.98, 1.04)	0.5364	1.01 (0.96, 1.05)	0.8268	0.96 (0.91, 1.01)	0.1090
WTI10	1.00 (1.00, 1.01)	0.0475	1.00 (0.99, 1.01)	0.2734	1.00 (0.98, 1.02)	0.8831
TyG-WC10	1.02 (1.01, 1.03)	0.0014	1.01 (0.99, 1.02)	0.5084	0.99 (0.97, 1.01)	0.2268
TyG–WHtR	1.54 (1.30, 1.83)	<0.0001	1.16 (0.89, 1.52)	0.2810	0.87 (0.60, 1.25)	0.4443
Hypertension						
TyG	1.87 (1.57, 2.21)	<0.0001	1.56 (1.20, 2.01)	0.0007	1.25 (0.89, 1.75)	0.2071
TyG-BMI10	1.05 (1.03, 1.07)	<0.0001	1.05 (1.02, 1.08)	0.0002	1.02 (0.99, 1.05)	0.0921
WTI10	1.01 (1.00, 1.01)	<0.0001	1.01 (1.00, 1.01)	0.0007	1.00 (0.99, 1.01)	0.0664
TyG-WC10	1.03 (1.02,	<0.0001	1.03 (1.01,	<0.0001	1.02 (1.00,	0.0064

	1.04)		1.04)		1.03)	
TyG–WHtR	1.81 (1.62, 2.03)	<0.0001	1.54 (1.30, 1.82)	<0.0001	1.32 (1.08, 1.62)	0.0062

Model I: No adjustment; Model II: Adjusted for age, sex, and race; Model III: Adjusted for age, sex, race, educational level, PIR, BUN, BUA, SCr, HDL-C, LDL-C, smoking status, alcohol consumption, hypertension and diabetes; TyG–WC10: triglyceride glucose–waist circumference divided by 10; TyG–WHtR: triglyceride glucose multiplied waist-to-height ratio.

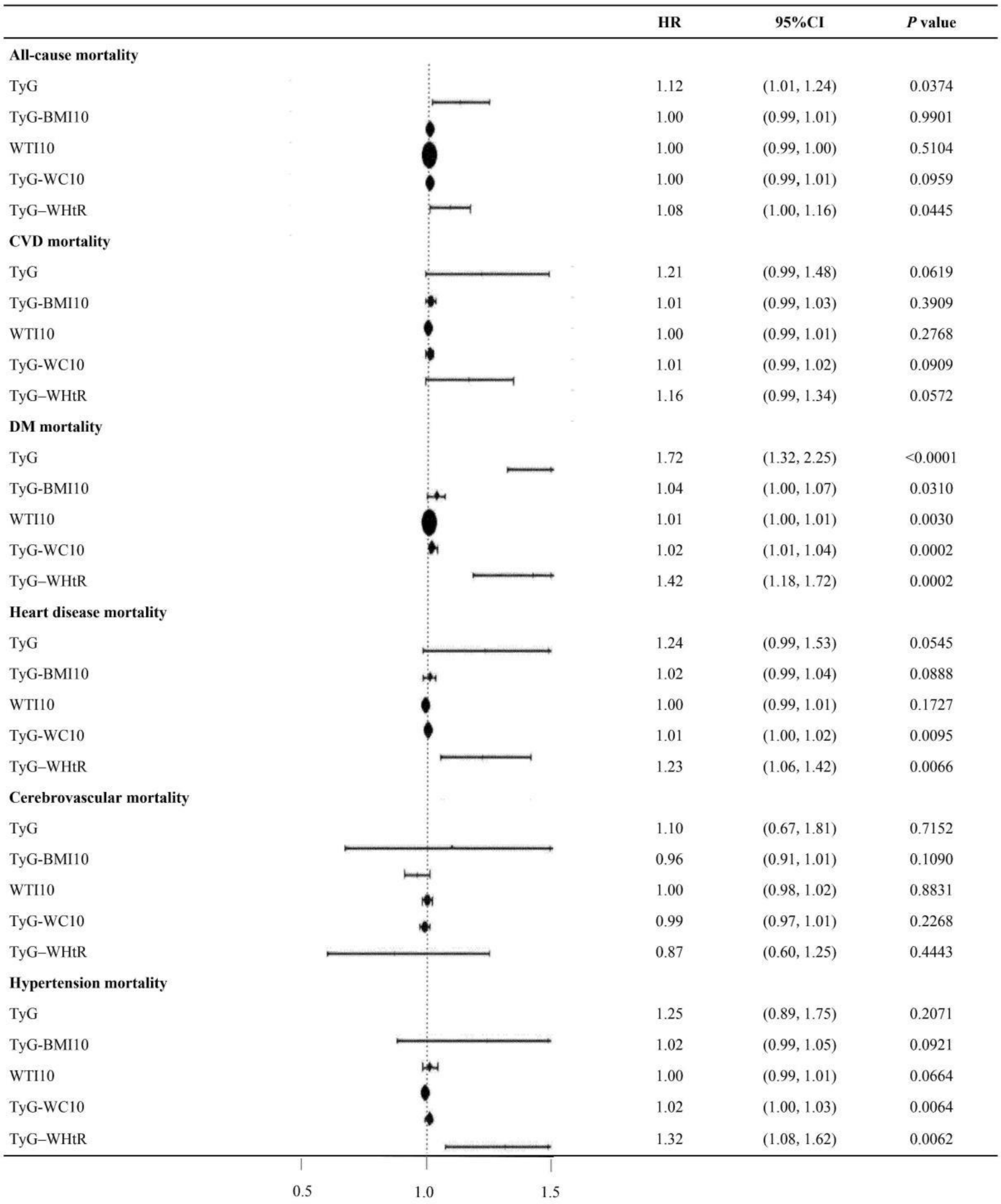

Supplementary Figure S6: Multivariable Cox regression analyses of TyG, TyG-BMI, WTI and modified TyG indices with all-cause, CVD, DM mortality and cause-specific cardiovascular disease mortality in patients with CKM syndrome stages 0-4.

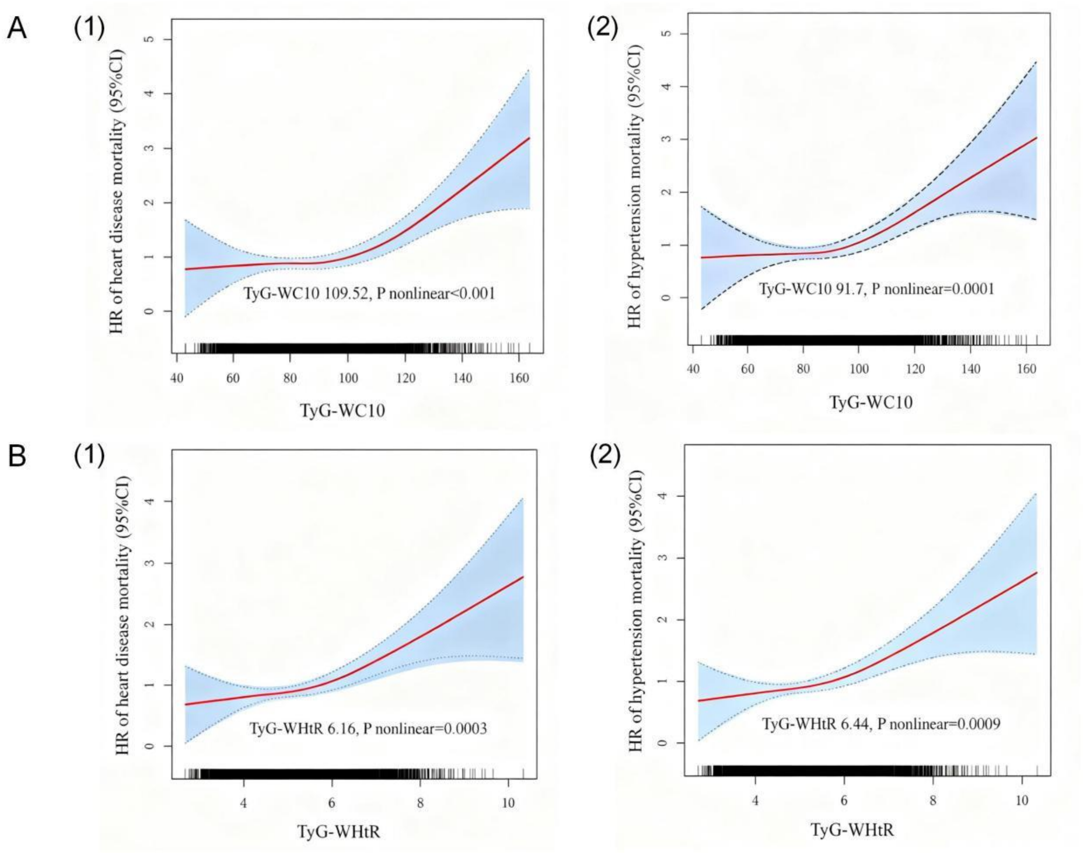

Supplementary Figure S7: Smooth curve fitting of the TyG–WC index (A) and TyG–BMI (B) with heart disease and hypertension mortality in patients with CKM syndrome stages 0-4.

Variable(s)	All-cause mortality HR（95%CI)	P	CVD mortality HR（95%CI)	P	DM mortality HR（95%CI)	P
<60						
TyG-WC10	1.02 (1.01, 1.03)	0.0001	1.03 (1.01, 1.04)	0.0002	1.04(1.02, 1.05)	<0.0001
TyG-WHtR	1.36 (1.16, 1.59)	0.0002	1.60 (1.26, 2.03)	0.0001	1.79 (1.40, 2.30)	<0.0001
≥60						
TyG-WC10	0.99 (0.98, 0.99)	0.0012	0.99 (0.98, 1.00)	0.1544	1.00 (0.99, 1.02)	0.3598
TyG-WHtR	0.92 (0.85, 0.99)	0.0492	0.96 (0.80, 1.15)	0.6500	1.11 (0.93, 1.32)	0.2656
Male						
TyG-WC10	1.01 (1.00, 1.02)	0.0059	1.01 (0.99, 1.02)	0.1193	1.03 (1.01, 1.04)	0.0005
TyG-WHtR	1.22 (1.08, 1.39)	0.0020	1.22 (0.98, 1.51)	0.0737	1.59 (1.28, 1.97)	<0.0001
Female						
TyG-WC10	0.99 (0.98, 1.00)	0.3628	1.01 (0.99, 1.02)	0.3399	1.02 (0.99, 1.04)	0.1141
TyG-WHtR	0.96 (0.85, 1.08)	0.4731	1.13 (0.90, 1.41)	0.2922	1.21 (0.87, 1.68)	0.2551
HBP (no)						
TyG-WC10	1.00 (0.99, 1.01)	0.9748	1.01 (0.99, 1.02)	0.5312	1.05 (1.02, 1.08)	0.0002
TyG-WHtR	1.03 (0.83, 1.29)	0.7627	1.14 (0.86, 1.50)	0.3658	2.47 (1.68, 3.65)	<0.0001
HBP(yes)						
TyG-WC10	1.00 (1.00, 1.01)	0.1098	1.01 (0.99, 1.02)	0.1782	1.02 (1.01, 1.04)	0.0036
TyG-WHtR	1.08 (0.99, 1.17)	0.0643	1.15 (0.97, 1.37)	0.1183	1.38 (1.09, 1.75)	0.0071
DM (no)						
TyG-WC10	1.00 (0.99, 1.01)	0.9693	1.00 (0.99, 1.01)	0.3489	1.04 (0.99, 1.08)	0.0619
TyG-WHtR	1.03 (0.92, 1.15)	0.6496	1.13 (0.94, 1.37)	0.1955	1.90 (0.98, 3.165)	0.0557
DM (yes)						
TyG-WC10	1.01 (1.00, 1.01)	0.0376	1.01 (0.99, 1.02)	0.1300	1.02 (1.00, 1.03)	0.0139
TyG-WHtR	1.11 (0.99, 1.25)	0.0652	1.11 (0.95, 1.30)	0.1820	1.27 (1.03, 1.56)	0.0234

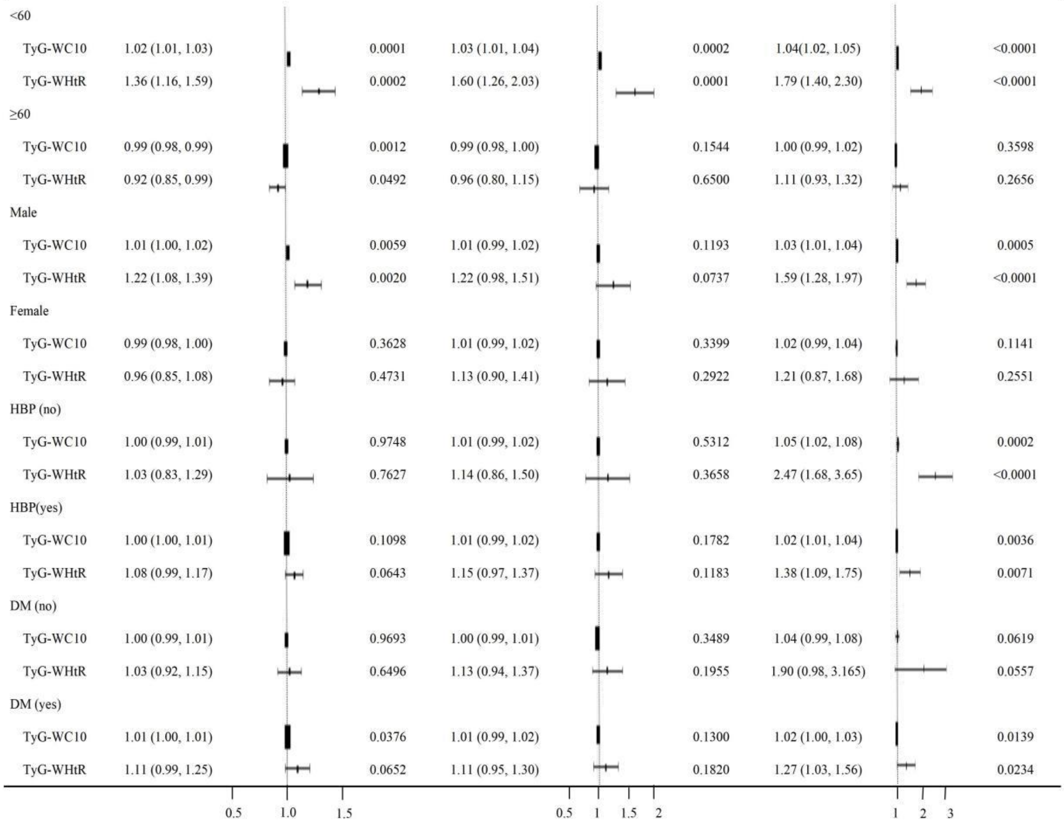

Supplementary Figure S8: Stratified analyses of modified TyG indices with all-cause, CVD and DM mortality across age, gender, HBP and DM in patients with CKM syndrome stages 0-4.
